# Supplementary material for: Bistable Forespore Engulfment in Bacillus subtilis by a Zipper Mechanism in Absence of the Cell Wall
Source: PLoS Comput Biol. 2014 Oct 30;10(10):e1003912. doi: 10.1371/journal.pcbi.1003912 (PMC4214620; doi:10.1371/journal.pcbi.1003912)
Supplement: Text S1 — Supporting information includes image analysis of cells lacking zipper proteins, details of numerical simulations, simulation parameters, and supplementary figures. (PDF) [file pcbi.1003912.s005.pdf]

Bistable forespore engulfment in *Bacillus subtilis* by a zipper mechanism in absence of the cell wall

Nikola Ojkic[1,2], Javier López-Garrido[3], Kit Pogliano[3], and Robert G. Endres[1,2]
[1] *Department of Life Sciences, Imperial College, London, United Kingdom,*
[2] *Centre for Integrative Systems Biology and Bioinformatics, Imperial College, London, United Kingdom,* [3] *Division of Biological Sciences, University of California at San Diego, La Jolla, California, USA*
(Dated: September 24, 2014)

Contents

1. Image analysis of mutants 1

2. Details of simulations 1
2.1. Model with energy balancing 1
2.2. Model with effective constraints 4
2.3. Simulation parameters 6

3. Supplementary figures 7

References 15

1. IMAGE ANALYSIS OF MUTANTS

We analyzed movies of cells lacking zipper proteins (SpoIIQ or SpoIIIAH) treated with lysozyme (see Fig. S1). Volume and surface area of mother and forespore cells over time were extracted using image analysis software JFilament (for details see Materials and Methods of the main text) [1]. Time point 0 minutes corresponds to mother-forespore cell separation (see Fig. 1B of the main text). 3D volume and surface area were calculated assuming rotational symmetry around the axis connecting center of masses of forespore and mother cells. Analysis was performed on previously published movies from [2]. We observed an increase in volume after cell-wall removal for both strains lacking one of the zipper proteins (see Fig. S1B) in contrast to WT cells shown in Fig. 2A cells for which a volume loss is observed. However, the surface area is unchanged (see Fig. S1C and D) similar to WT cells (see Fig. 2B).

2. DETAILS OF SIMULATIONS

In the main text we used a Langevin equation to simulate dynamics of the mother cell in 3D assuming rotational symmetry around the axis connecting the centers of masses of the mother cell and forespore. We explored two models that only differ in the applied constraints on the mother-cell membrane. In the first model, the surface tension and pressure difference between inner and outer medium realistically balance each other (see Results of the main text). In the second model, effective constraints are artificially introduced on the mother-cell volume and surface area (see Discussion in the main text). These two models are explained in the two following sections.

2.1. Model with energy balancing

Here we explain the main model from the main text in which surface tension (σ_1 and σ_2) and turgor pressure (Δp) balance each other. The turgor pressure is the osmotic pressure difference between inner and outer medium across

the mother-cell membrane [3, 4]. Parameters σ_1 and Δp are treated as independent, while σ_2 is adjusted to stabilize the initially round mother cell. Langevin equation is:

$$\zeta_i \frac{\mathrm{d}\mathbf{R}_i}{\mathrm{d}t} = \mathbf{F}_i^{\mathrm{bend}} + \mathbf{F}_i^{\mathrm{stoch}} + \mathbf{F}_i^{\mathrm{QAH}} + \mathbf{F}_i^{\sigma} + \mathbf{F}_i^{\Delta p}. \tag{1}$$

On the left-hand side, ζ_i is the drag coefficient of the ith bead representing a ribbon of length $L_i = 2\pi x_i$ and width l_0 (see Fig. 3 of the main text). We used $\zeta_i \approx 4\pi\eta_{\mathrm{med}} L_i$ [5], where η_{med} is the effective medium viscosity. On the right-hand side of Eq. 1 we have contributions of membrane bending, stochastic fluctuations, zipper binding of mother cell and forespore membranes, surface tension, and difference in osmotic pressure across the membrane, respectively. The forces are defined as follows.

Membrane bending: The bending energy corresponding to the unit area of the ith ribbon is:

$$E_i^{\mathrm{bend}} = \frac{1}{2}\kappa_{\mathrm{b}}(c_{\mathrm{m},i} + c_{\mathrm{p},i})^2\, \Delta A_i, \tag{2}$$

where $\Delta A_i = 2\pi x_i l_0$ is the surface area of the ribbon, $c_{\mathrm{m},i} \equiv \mathrm{d}\theta_i/\mathrm{d}s$ is the meridian principle curvature, $c_{\mathrm{p},i} \equiv \sin\theta_i/x_i$ is the principle curvature along the parallels (see Fig. 3 of the main text) [4], l_0 is the distance between two neighboring beads, κ_{b} is the membrane bending rigidity, and θ_i is the angle between unit normal vector $\hat{n}_i$ of the contour and z-axis. A Gaussian curvature contribution is neglected as the topology of the mother-cell membrane does not change during engulfment [6]. Summing Eq. 2 over the whole surface area we obtain the total bending energy:

$$E_{\mathrm{tot}}^{\mathrm{bend}} = \pi\kappa_{\mathrm{b}} l_0 \sum_{j=1}^{N} x_j (c_{\mathrm{m},j} + c_{\mathrm{p},j})^2, \tag{3}$$

where N is the total number of beads. The force due to bending is determined by the negative energy gradient:

$$\mathbf{F}_i^{\mathrm{bend}} = -\frac{\partial E_{\mathrm{tot}}^{\mathrm{bend}}}{\partial \mathbf{R}_i}. \tag{4}$$

Therefore, the x and z components of the bending force are:

$$F_{i,x}^{\mathrm{bend}} = -\pi\kappa_{\mathrm{b}} l_0 \left[(c_{\mathrm{m},i} + c_{\mathrm{p},i})^2 + 2\sum_{j=1}^{N} x_j (c_{\mathrm{m},j} + c_{\mathrm{p},j}) \left(\frac{\partial c_{\mathrm{m},j}}{\partial x_i} + \frac{\partial c_{\mathrm{p},j}}{\partial x_i} \right) \right], \tag{5}$$

$$F_{i,z}^{\mathrm{bend}} = -2\pi\kappa_{\mathrm{b}} l_0 \sum_{j=1}^{N} x_j (c_{\mathrm{m},j} + c_{\mathrm{p},j}) \left(\frac{\partial c_{\mathrm{m},j}}{\partial z_i} + \frac{\partial c_{\mathrm{p},j}}{\partial z_i} \right). \tag{6}$$

Using the definitions of the principle curvatures introduced above, we have:

$$c_{\mathrm{m},i} \equiv \frac{\mathrm{d}\theta_i}{\mathrm{d}s} = \frac{|\mathrm{d}\hat{\mathbf{t}}_i|}{\mathrm{d}s} = \frac{|\hat{\mathbf{t}}_{i+1} - \hat{\mathbf{t}}_i|}{|\mathbf{R}_{i+1} - \mathbf{R}_i|} = \frac{\sqrt{2(1 - \hat{\mathbf{t}}_{i+1}\hat{\mathbf{t}}_i)}}{|\mathbf{R}_{i+1} - \mathbf{R}_i|}, \tag{7}$$

$$c_{\mathrm{p},i} \equiv \frac{\sin\theta_i}{x_i} = \frac{\hat{z}\,\hat{\mathbf{t}}_i}{x_i}, \tag{8}$$

where $\hat{\mathbf{t}}_i \equiv (\mathbf{R}_{i+1} - \mathbf{R}_i)/|\mathbf{R}_{i+1} - \mathbf{R}_i|$ is the unit tangent vector. Using Eqs. 7, 8, and the following identity [7]:

$$\frac{\partial \hat{\mathbf{t}}_i}{\partial \mathbf{R}_j} = \frac{1}{l_0}(\delta_{i+1,j} - \delta_{i,j})(\hat{\mathbf{I}} - \hat{\mathbf{t}}_i\hat{\mathbf{t}}_i^{\mathrm{T}}), \tag{9}$$

where $\delta_{i,j}$ is the Kroneker symbol, $\hat{\mathbf{I}}$ is the unit matrix, and

$$\hat{\mathbf{t}}_i\hat{\mathbf{t}}_i^{\mathrm{T}} \equiv \begin{pmatrix} t_{i,x}^2 & t_{i,x}t_{i,z} \\ t_{i,x}t_{i,z} & t_{i,z}^2 \end{pmatrix}, \tag{10}$$

the expressions for the bending force become:

$$\begin{aligned}
F_{i,x}^{\mathrm{bend}} = -2\pi\kappa_{\mathrm{b}} l_0 \Bigg[& \frac{1}{2}(c_{\mathrm{m},i}+c_{\mathrm{p},i})^2 - \frac{\sqrt{2}}{2l_0^2}\frac{x_{i-2}(c_{\mathrm{m},i-2}+c_{\mathrm{p},i-2})}{\sqrt{1-\hat{\mathbf{t}}_{i-2}\hat{\mathbf{t}}_{i-1}}}(t_{i-2,x}-t_{i-1,x}\hat{\mathbf{t}}_{i-2}\hat{\mathbf{t}}_{i-1}) + \\
& \frac{\sqrt{2}}{2l_0^2}\frac{x_{i-1}(c_{\mathrm{m},i-1}+c_{\mathrm{p},i-1})}{\sqrt{1-\hat{\mathbf{t}}_{i-1}\hat{\mathbf{t}}_{i}}}(t_{i-1,x}-t_{i,x}\hat{\mathbf{t}}_{i-1}\hat{\mathbf{t}}_{i}) - \\
& \frac{\sqrt{2}}{2l_0^2}\frac{x_{i-1}(c_{\mathrm{m},i-1}+c_{\mathrm{p},i-1})}{\sqrt{1-\hat{\mathbf{t}}_{i-1}\hat{\mathbf{t}}_{i}}}(t_{i,x}-t_{i-1,x}(3\hat{\mathbf{t}}_{i-1}\hat{\mathbf{t}}_{i}-2)) + \\
& \frac{\sqrt{2}}{2l_0^2}\frac{x_{i}(c_{\mathrm{m},i}+c_{\mathrm{p},i})}{\sqrt{1-\hat{\mathbf{t}}_{i+1}\hat{\mathbf{t}}_{i}}}(t_{i+1,x}-t_{i,x}(3\hat{\mathbf{t}}_{i+1}\hat{\mathbf{t}}_{i}-2)) - \\
& \frac{(c_{\mathrm{m},i}+c_{\mathrm{p},i})}{x_i}t_{i,z} - \frac{(c_{\mathrm{m},i-1}+c_{\mathrm{p},i-1})}{l_0}t_{i-1,x}t_{i-1,z} + \frac{(c_{\mathrm{m},i}+c_{\mathrm{p},i})}{l_0}t_{i,x}t_{i,z}\Bigg],
\end{aligned} \tag{11}$$

$$\begin{aligned}
F_{i,z}^{\mathrm{bend}} = 2\pi\kappa_{\mathrm{b}} l_0 \Bigg[& \frac{\sqrt{2}}{2l_0^2}\frac{x_{i-2}(c_{\mathrm{m},i-2}+c_{\mathrm{p},i-2})}{\sqrt{1-\hat{\mathbf{t}}_{i-2}\hat{\mathbf{t}}_{i-1}}}(t_{i-2,z}-t_{i-1,z}\hat{\mathbf{t}}_{i-2}\hat{\mathbf{t}}_{i-1}) - \\
& \frac{\sqrt{2}}{2l_0^2}\frac{x_{i-1}(c_{\mathrm{m},i-1}+c_{\mathrm{p},i-1})}{\sqrt{1-\hat{\mathbf{t}}_{i-1}\hat{\mathbf{t}}_{i}}}(t_{i-1,z}-t_{i,z}\hat{\mathbf{t}}_{i-1}\hat{\mathbf{t}}_{i}) + \\
& \frac{\sqrt{2}}{2l_0^2}\frac{x_{i-1}(c_{\mathrm{m},i-1}+c_{\mathrm{p},i-1})}{\sqrt{1-\hat{\mathbf{t}}_{i-1}\hat{\mathbf{t}}_{i}}}(t_{i,z}-t_{i-1,z}(3\hat{\mathbf{t}}_{i-1}\hat{\mathbf{t}}_{i}-2)) - \\
& \frac{\sqrt{2}}{2l_0^2}\frac{x_{i}(c_{\mathrm{m},i}+c_{\mathrm{p},i})}{\sqrt{1-\hat{\mathbf{t}}_{i+1}\hat{\mathbf{t}}_{i}}}(t_{i+1,z}-t_{i,z}(3\hat{\mathbf{t}}_{i+1}\hat{\mathbf{t}}_{i}-2)) - \\
& \frac{(c_{\mathrm{m},i-1}+c_{\mathrm{p},i-1})}{l_0}(1-t_{i-1,z}^2) + \frac{(c_{\mathrm{m},i}+c_{\mathrm{p},i})}{l_0}(1-t_{i,z}^2)\Bigg].
\end{aligned} \tag{12}$$

Stochastic force: The stochastic force due to thermal noise is defined as [7]:

$$\langle \mathbf{F}_i^{\mathrm{stoch}} \mathbf{F}_i^{\mathrm{stoch\,T}} \rangle = \frac{2k_{\mathrm{B}}T\zeta_0(\frac{L_i}{l_0})^2}{\Delta t}\hat{\mathbf{I}}, \tag{13}$$

with $k_{\mathrm{B}}T$ the thermal energy, Δt the simulation time step, and ζ_0 is the frictional coefficient of segment with length l_0, and $\hat{\mathbf{I}}$ is the unit matrix. We introduced the $(L_i/l_0)^2$ correction to the thermal force to make displacements due to the thermal noise independent of position x_i and z_i. Hence all beads fluctuate with average zero displacement and force variance given by Eq. 13.

Binding of mother-cell and forespore membranes: Forespore protein SpoIIQ and mother-cell protein SpoIIIAH proteins bind with high binding energy (see Table S1) [8]. We modeled the protein-protein interaction as a simple elastic spring:

$$\mathbf{F}_i^{\mathrm{QAH}} = -k_{\mathrm{QAH}} \cdot (|\mathbf{R}_i - \mathbf{R}_{\mathrm{fspore}}| - l_{\mathrm{QAH}}) \cdot \mathrm{H}(l_{\mathrm{QAH}} - |\mathbf{R}_i - \mathbf{R}_{\mathrm{fspore}}|) \tag{14}$$

Vector $\mathbf{R}_{\mathrm{fspore}}$ points toward the position of the forespore contour closest to the mother's i^{th} bead, l_{QAH} is the interaction distance between SpoIIQ-SpoIIIAH, $k_{\mathrm{QAH}} = 2E_{\mathrm{QAH}}/l_{\mathrm{QAH}}^2$ is the spring constant of SpoIIQ-SpoIIIAH interaction, and H( ) is the Heaviside step function preventing connections with distances larger than l_{QAH}.

The last two terms of Eq. 1 are derived from energy

$$\tilde{E} \equiv E^{\sigma} + E^{\Delta p} = \sigma_1 S(1+\sigma_2 S) - \Delta p V, \tag{15}$$

where the positive surface-tension term tries to deflate the mother cell while the negative pressure term tries to inflate the mother cell. The non-linear term $\sigma_1\sigma_2 S^2$ is introduced to stabilize the mother cell [9]. In particular, for given σ_1

and Δp, parameter σ_2 is calculated to equilibrate the initial round mother cell (with experimentally observed volume V_0) allowing the exploration of parameter values (σ_1 and Δp) independently of each other. The initial mother-cell is represented by a sphere of volume $V_0 = S_0^{\frac{3}{2}}/(6\sqrt{\pi})$ prior to engulfment (Table S1). Parameter σ_2 is found by imposing stability:

$$\left.\frac{\partial \tilde{E}}{\partial S}\right|_{S=S_0} = \left.\frac{\partial \tilde{E}}{\partial V}\right|_{V=V_0} = 0. \tag{16}$$

Solving Eq. 16 for σ_2 we obtain:

$$\sigma_2 = \frac{\Delta p}{8\sigma_1\sqrt{\pi S_0}} - \frac{1}{2S_0}. \tag{17}$$

The stable equilibrium is obtained from the condition $\left.\frac{\partial^2 \tilde{E}}{\partial S^2}\right|_{S=S_0} > 0$:

$$\Delta p > \frac{8\sqrt{\pi}}{\sqrt{S_0}}\sigma_1 = \frac{4\sigma_1}{R_0}, \tag{18}$$

where the R_0 is the initial mother-cell radius and the right-hand side is Laplace law.

Surface-tension force: From the Eq. 15 we derive surface tension force for the ith bead as follows:

$$\mathbf{F}_i^{\sigma} = -\frac{\partial E^{\sigma}}{\partial \mathbf{R}_i} = -\frac{\partial}{\partial \mathbf{R}_i}\Big[\sigma_1 S(1+\sigma_2 S)\Big] = -\sigma_1(1+2\sigma_2 S)\frac{\partial S}{\partial \mathbf{R}_i}. \tag{19}$$

Here, $S = \sum_{i=1}^{N} 2\pi x_i\, l_{0,i}$ and $l_{0,i} = \sqrt{(x_{i+1}-x_i)^2 + (z_{i+1}-z_i)^2}$. Therefore, the x and z components of the surface tension force are:

$$F_{i,x}^{\sigma} = -2\pi\sigma_1(1+2\sigma_2 S)(l_0 + x_{i-1}t_{i-1,x} - x_i t_{i,z}); \tag{20}$$
$$F_{i,z}^{\sigma} = -2\pi\sigma_1(1+2\sigma_2 S)(x_{i-1}t_{i-1,z} - x_i t_{i,z}). \tag{21}$$

Turgor-pressure force: From the Eq. 15 we derive turgor pressure force for the ith bead as follows:

$$\mathbf{F}_i^{\Delta p} = -\frac{\partial E^{\Delta p}}{\partial \mathbf{R}_i} = -\frac{\partial}{\partial \mathbf{R}_i}(-\Delta p V), \tag{22}$$

with $V = \sum_{i=1}^{N} \pi x_i^2\, l_{0,i}\hat{\mathbf{t}}_i\hat{z}$. Therefore, the x and z components of the turgor pressure force are:

$$F_{i,x}^{\Delta p} = 2\pi\,\Delta p\, l_0\, x_i\, t_{i,z}, \tag{23}$$
$$F_{i,z}^{\Delta p} = \pi\,\Delta p\,(x_{i-1}^2 - x_i^2). \tag{24}$$

This model accounts for realistic balancing of surface tension (contraction) and osmotic pressure (expansion).

2.2. Model with effective constraints

Similar to the model with energy balancing given by Eq. 1, the Langevin equation of the overdamped dynamics of the ith bead at the position $\mathbf{R}_i$ is given by:

$$\zeta_i\frac{\mathrm{d}\mathbf{R}_i}{\mathrm{d}t} = \mathbf{F}_i^{\text{bend}} + \mathbf{F}_i^{\text{stoch}} + \mathbf{F}_i^{\text{QAH}} + \mathbf{F}_i^{S} + \mathbf{F}_i^{V}, \tag{25}$$

In this equation the first three terms on the right-hand side are the same as in Eq. 1. However, the last two terms are given as follows.

Surface-area constraint: An effective surface-area constraint is introduced to keep the surface area of the mother cell close to a preferred initial surface area S_0 [10]:

$$\mathbf{F}_i^S = -\frac{\partial}{\partial \mathbf{R}_i}\left[\frac{k_S}{2}(S - S_0)^2\right], \tag{26}$$

Therefore, the x and z components of the surface force are:

$$F_{i,x}^S = -2\pi k_S(S - S_0)(l_0 + x_{i-1}t_{i-1,x} - x_i t_{i,z}), \tag{27}$$

$$F_{i,z}^S = -2\pi k_S(S - S_0)(x_{i-1}t_{i-1,z} - x_i t_{i,z}). \tag{28}$$

Volume constraint: An effective volume constraint is introduced using a harmonic potential to keep the mother-cell volume close to a preferred initial volume V_0 [10, 11] :

$$\mathbf{F}_i^V = -\frac{\partial}{\partial \mathbf{R}_i}\left[\frac{k_V}{2}(V - V_0)^2\right]. \tag{29}$$

Therefore, the x and z components of the volume force are:

$$F_{i,x}^V = -2\pi l_0 x_i k_V(V - V_0)t_{i,z}, \tag{30}$$

$$F_{i,z}^V = -\pi k_V(V - V_0)(x_{i-1}^2 - x_i^2). \tag{31}$$

This model with the artificial volume and surface-area constraints allows the independent exploration of their effects on engulfment (see Discussion in the the main text).

2.3. Simulation parameters

Parameters used in simulations can be found in Table S1. The effective temperature was determined in Fig. S2B. Parameters (σ_1 and Δp) of the first model are given after the main simulation parameters, followed by parameters of the second model (k_S and k_V).

TABLE I: Model parameters

Symbol	Physical quantity	Values used in simulation	Sources / References	Notes
$V_0 = V_{\rm m}$	Initial mother volume	2 μm^3	Analyzed data from [2]	
$S_0 = S_{\rm m}$	Initial mother-surface area	7.6 μm^2	Calculated from V_0	
R_0	Initial mother-cell radius	0.782 μm	Calculated from V_0	
$V_{\rm f}$	Initial forespore volume	0.2 μm^3	Analyzed data from [2]	
$S_{\rm f}$	Initial forespore surface area	2 μm^2	Calculated from $V_{\rm f}$	
T_0	Room temperature	300 K		
$\kappa_{\rm b}$	Membrane bending rigidity	10 $k_{\rm B}T_0$, (1-30)$k_{\rm B}T_0$	[6]	
c_0	Intrinsic curvature	(-120 - 20) μm $^{-1}$	Our simulations (Fig. S7)	
σ	Surface tension	(1-100) pN/μm	[9, 12, 13]	
$\eta_{\rm wat}$	Water viscosity	0.001 Pa s	[6]	
$\eta_{\rm med}$	Medium viscosity	1.3 Pa s	[14]	
$E_{\rm QAH}$	SpoIIQ-SpoIIIAH binding energy	-64 $k_{\rm B}T_0$	[8]	
$l_{\rm QAH}$	SpoIIQ-SpoIIIAH interaction distance	0.01 μm	[15, 16]	
l_0	Mesh size	0.01 μm		Our simulations
dt	Time step	0.5 μs		Our simulations
σ_1	Surface tension	(1-100) pN/μm	See above	Model 1
Δp	Osmotic pressure	(1-1000) Pa		Model 1
k_S	Explicit surface-area constraint	(0-1) nN/μm^3		Model 2
k_V	Explicit volume constraint	(0-10) nN/μm^5		Model 2

3. SUPPLEMENTARY FIGURES

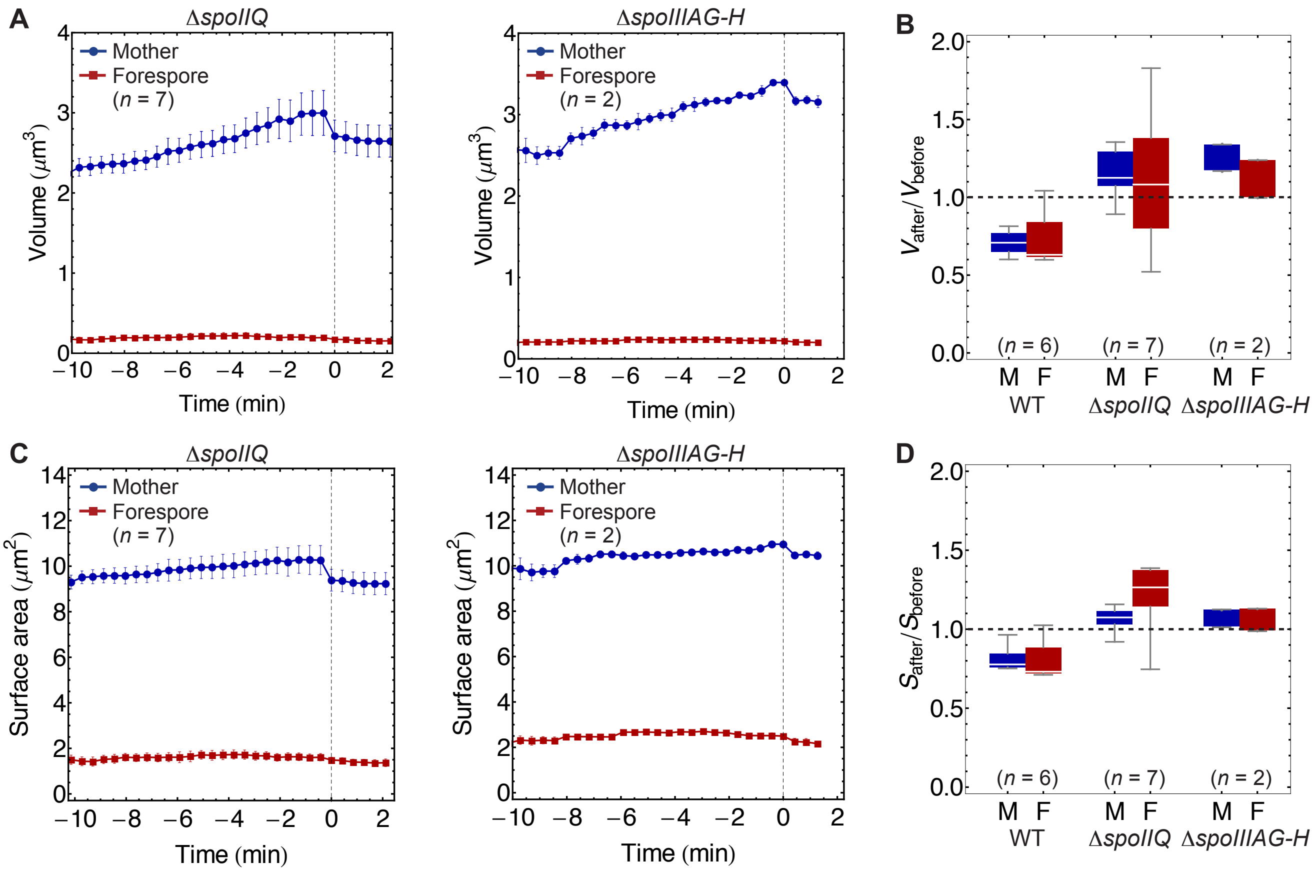

FIG. 1: **Image analysis of Δ*spoIIQ* and Δ*spoIIIAG-H* cells treated with cell-wall removal enzyme.** (A-D) Volume and surface area over time for mother and forespore cells as determined by active contours (for details see Materials and Methods of the main text). Time 0 minutes corresponds to mother-forespore cell separation (see Fig. 1B of the main text). 3D volume and surface area were calculated assuming rotational symmetry around the axis connecting center of masses of mother cell and forespore. All analysis were performed on previously published movies from [2]. (A) Volume vs time for Δ*spoIIQ* (left) and Δ*spoIIIAG-H* (right) cells. (B) Box-and-whisker plot of volume after ($V_{\rm after}$) divided by volume before ($V_{\rm before}$) cell-wall removal. Different strains are indicated. Mother-cell volume loss occurred only in WT cells (without cell wall). (C and D) Similar as in A and B. Minor mother-cell surface area loss (∼ 5-15 %) occurred only in WT cells. Parameter n indicates number of cells analyzed.

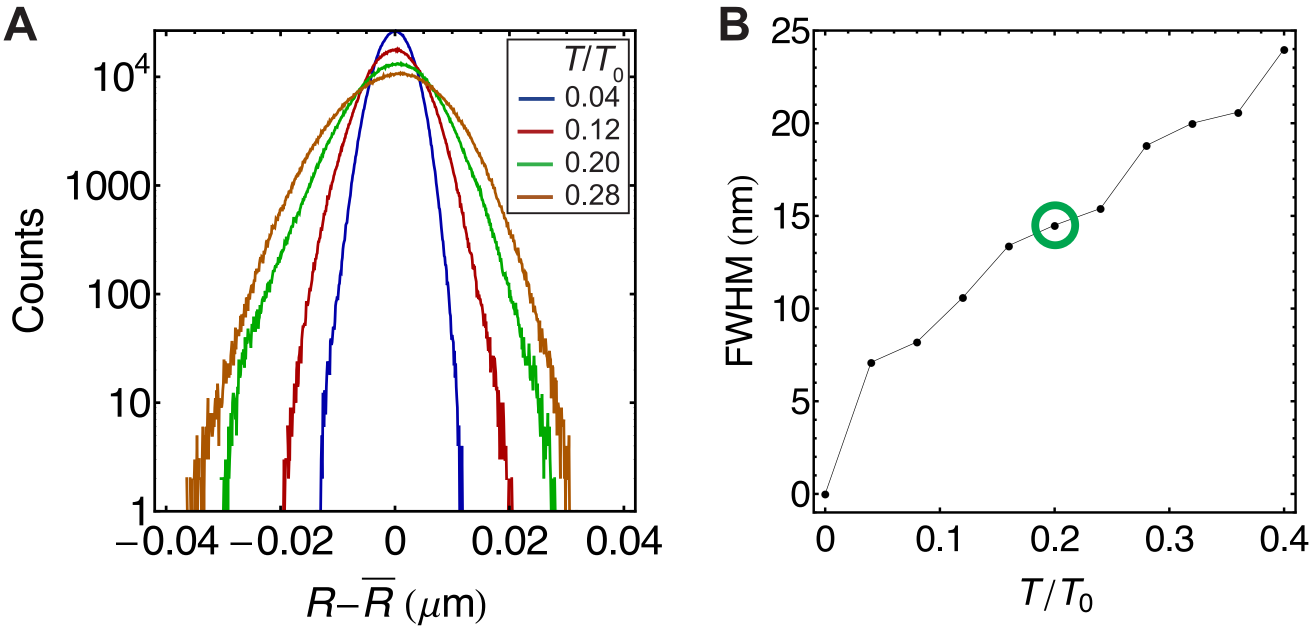

FIG. 2: **Determination of effective temperature.** (A-B) Effective temperature was chosen so that typical amplitudes of membrane fluctuations due to thermal noise are $\sim$ 15 nm as experimentally observed in other systems [17, 18]. Parameters used in simulations: $\sigma_1 = 50\ pN/\mu$m, $\Delta p = 850$ Pa, $\kappa_\mathrm{b} = 10\ k_\mathrm{B}T_0$. (A) Histogram of radii collected for all bead positions (initial total number of beads $N = 490$) every 2.5 ms, for first 10 s of simulations. Different colors represent different simulations with corresponding effective temperatures (see Eq. 13). (B) Full width at half maximum (FWHM) versus effective temperature. We chose $T/T_0 = 0.2$ for all other simulations in main and supplementary text causing typical ripple size in radial direction of $\sim$ 15 nm for this set of σ_1 and Δp parameters. Simulations of Fig. 5 of the main text have typical ripple size of $\sim$ 15 nm.

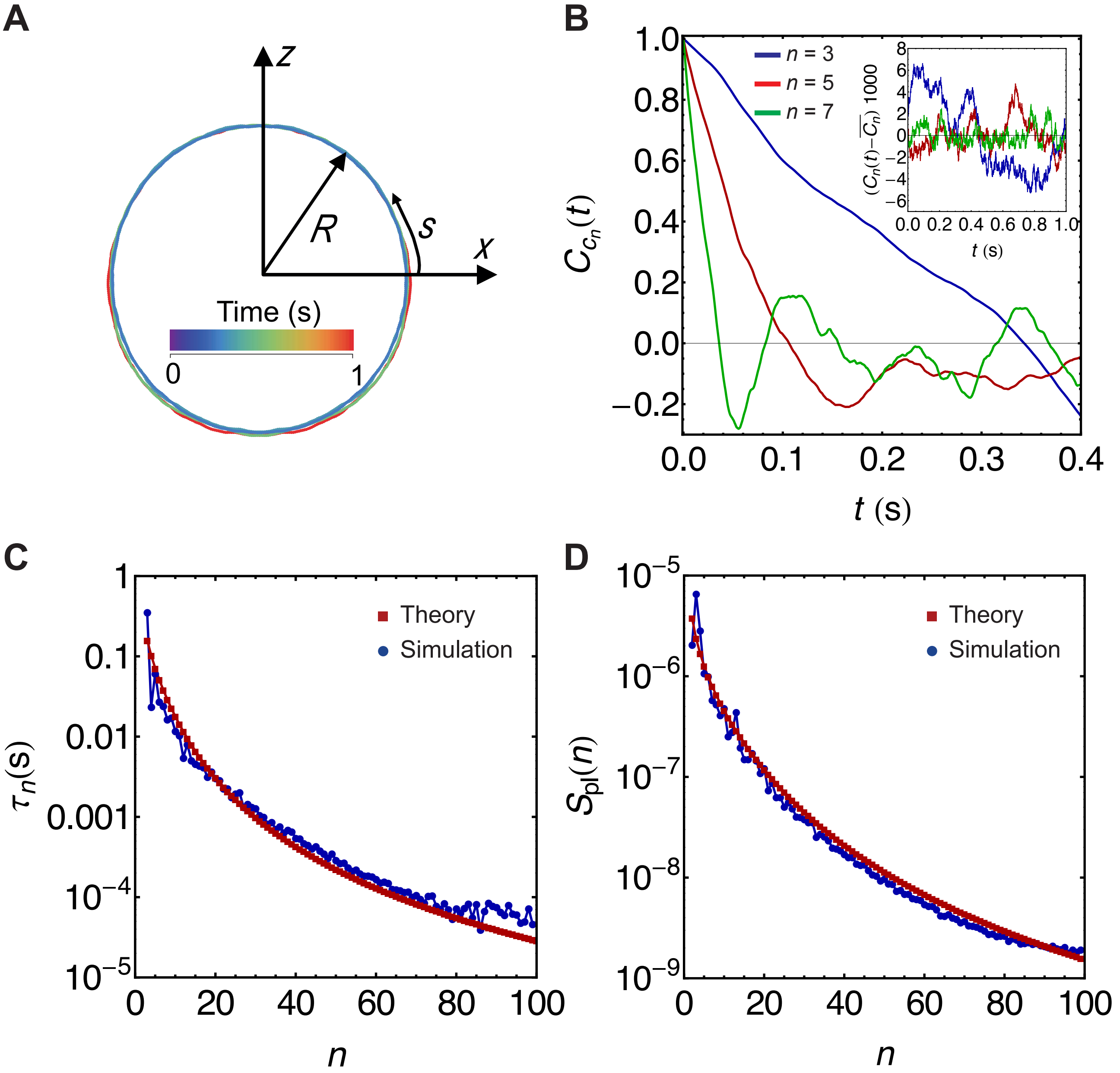

FIG. 3: **Model validation and simulation results of fluctuating round mother cell with thermal noise.** (A-D) Space Fourier coefficients of membrane contours were collected ($c_n \equiv |\tilde{c}_n|$). Parameters used in simulation: $\sigma_1 = 50\ pN/\mu$m, $\Delta p =$ 850 Pa, $\kappa_{\rm b} = 20\ k_{\rm B}T_0$, $\langle R \rangle = 0.782\ \mu$m. (B, Inset) Three examples of Fourier coefficients versus time. (B) Three examples of autocorrelation function versus time for data shown in the inset. Since $C_{c_n} \sim e^{-t/\tau_n}$ [19], relaxation times (τ_n) are obtained as fitted parameters. (C) Relaxation times versus Fourier modes. Theoretical expressions are given in Materials and Methods of the main text [20, 21]. Parameter $\tilde{\sigma} = 50$. (D) For collection of 6000 contours obtained during first 1.5 s of thermal equilibrium simulations, dimensionless fluctuation spectrum ($S_{\rm pl}(n) \equiv \langle c_n^2 \rangle - \langle c_n \rangle^2$) was plotted along with theoretical expression for planer membranes from [12]. For analytical results for τ_n and $S_{\rm pl}(n)$, see Materials and Methods of the main text.

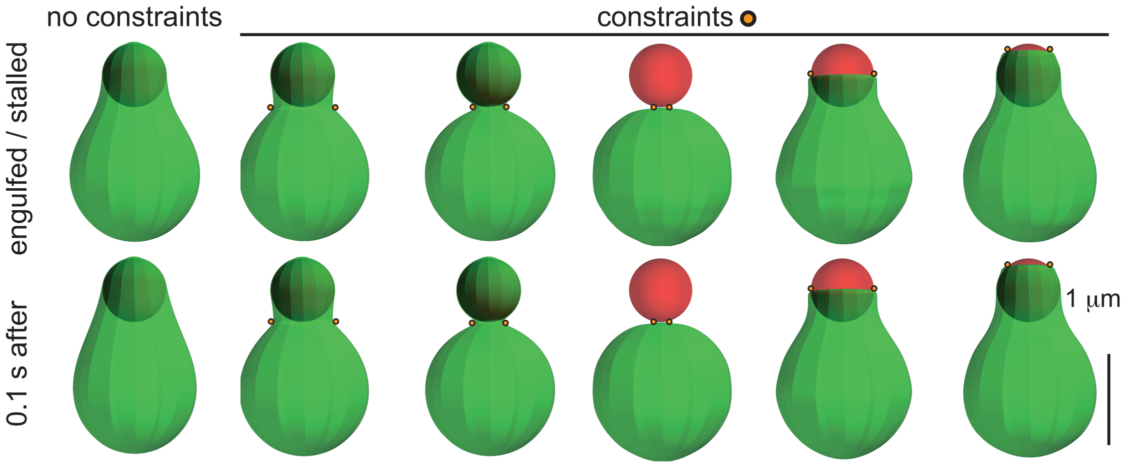

FIG. 4: **Effect of cell-wall remnants on engulfment.** Cell-wall remnants are simulated as beads of size l_0 with repulsive excluded volume interactions. Cell-wall remnants in the neck region can contribute to snowman-like shapes (left). Cell-wall remnants can stall progression of engulfing membranes (right).

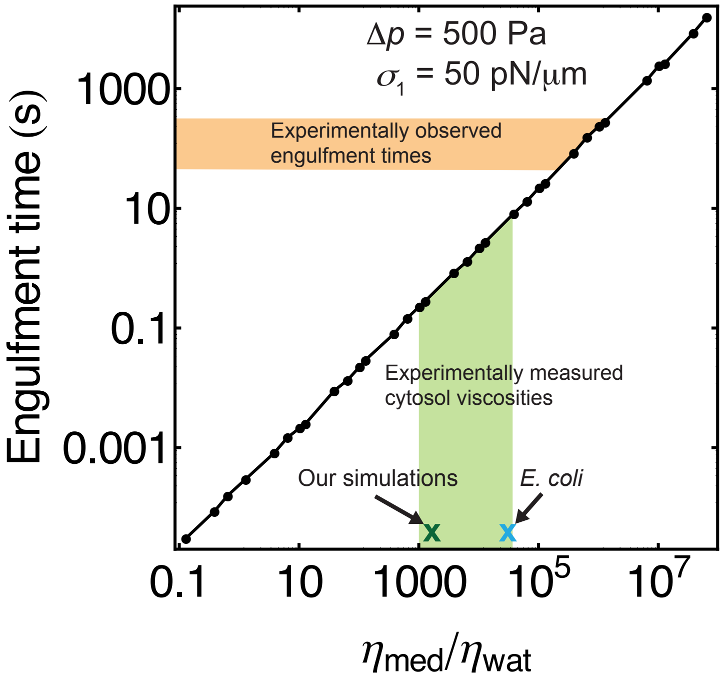

FIG. 5: **Relation between medium viscosity and engulfment time.** We simulated engulfment for various medium viscosities (η_{med}) and extracted engulfment times. All other parameters were fixed ($\sigma_1 = 50$ pN/μm, $\Delta p = 500$ Pa, $\rho = 10^4 \mu\text{m}^{-2}$). Engulfment times increased linearly with η_{med}, while cup morphologies remained the same. Green region represents experimentally measured cytosol viscosities [14]. Crosses mark medium viscosity used in our simulations (dark green) and measured viscosity in *E. coli* (blue) [22]. Orange region represents experimentally observed engulfment times. For the experimentally measured viscosities, engulfment times are approximately an order of magnitude smaller than the experimentally observed engulfment times.

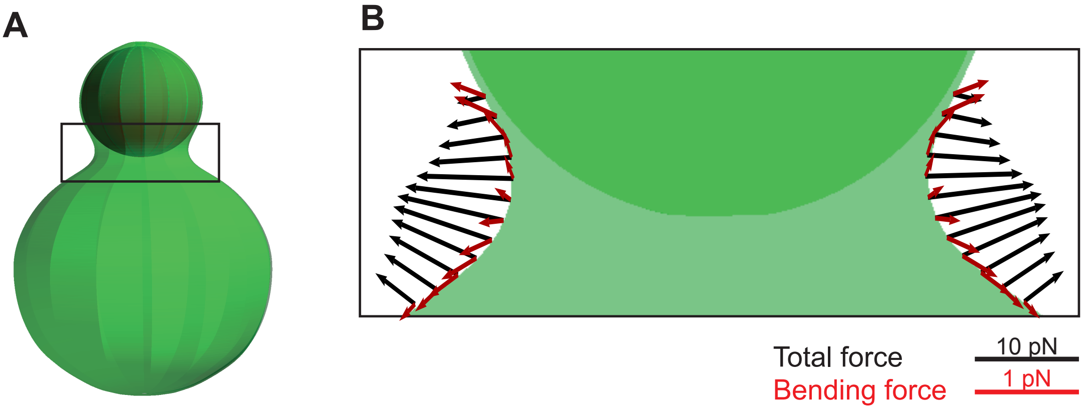

FIG. 6: **Neck-region forces.** (A) Snapshot of engulfed forespore (σ_1 = 50 pN/μm, Δp = 200 Pa, κ_b = 10 k_BT_0). Once forespore is engulfed the stochastic thermal force was turned off in Eq. 1. (B) Subsequent total forces and bending forces on beads in neck region during equilibration with scale bars as indicated.

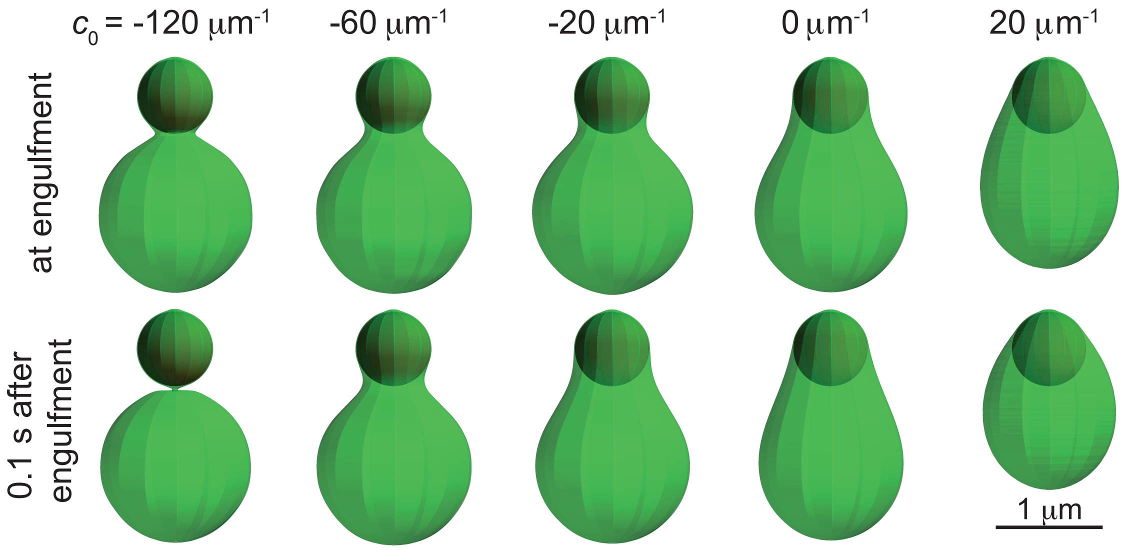

FIG. 7: **Effect of intrinsic membrane curvature on engulfment.** In simulations with intrinsic curvatures ($c_0 \neq 0$) the bending energy is defined as $E_i^{\text{bend}} = \frac{1}{2}\kappa_b(c_{m,i} + c_{p,i} - c_0)^2\,\Delta A_i$ [6], similar to Eq. 2. Parameters used in simulations: σ_1 = 50 pN/μm, Δp = 500 Pa, κ_b = 10 k_BT_0. Once forespores are engulfed (first row) membrane fluctuations were turned off in simulations. Subsequent equlibration without fluctuations was examined after 0.1 s (second row). Successful engulfment were observed for (-120 $\leqslant c_0 \leqslant$ 20) μm^{-1}. Extreme high negative curvatures produce high outward force in the neck region preventing further engulfment; extreme high positive curvatures stall engulfment by preventing leading-edge fluctuations (not shown).

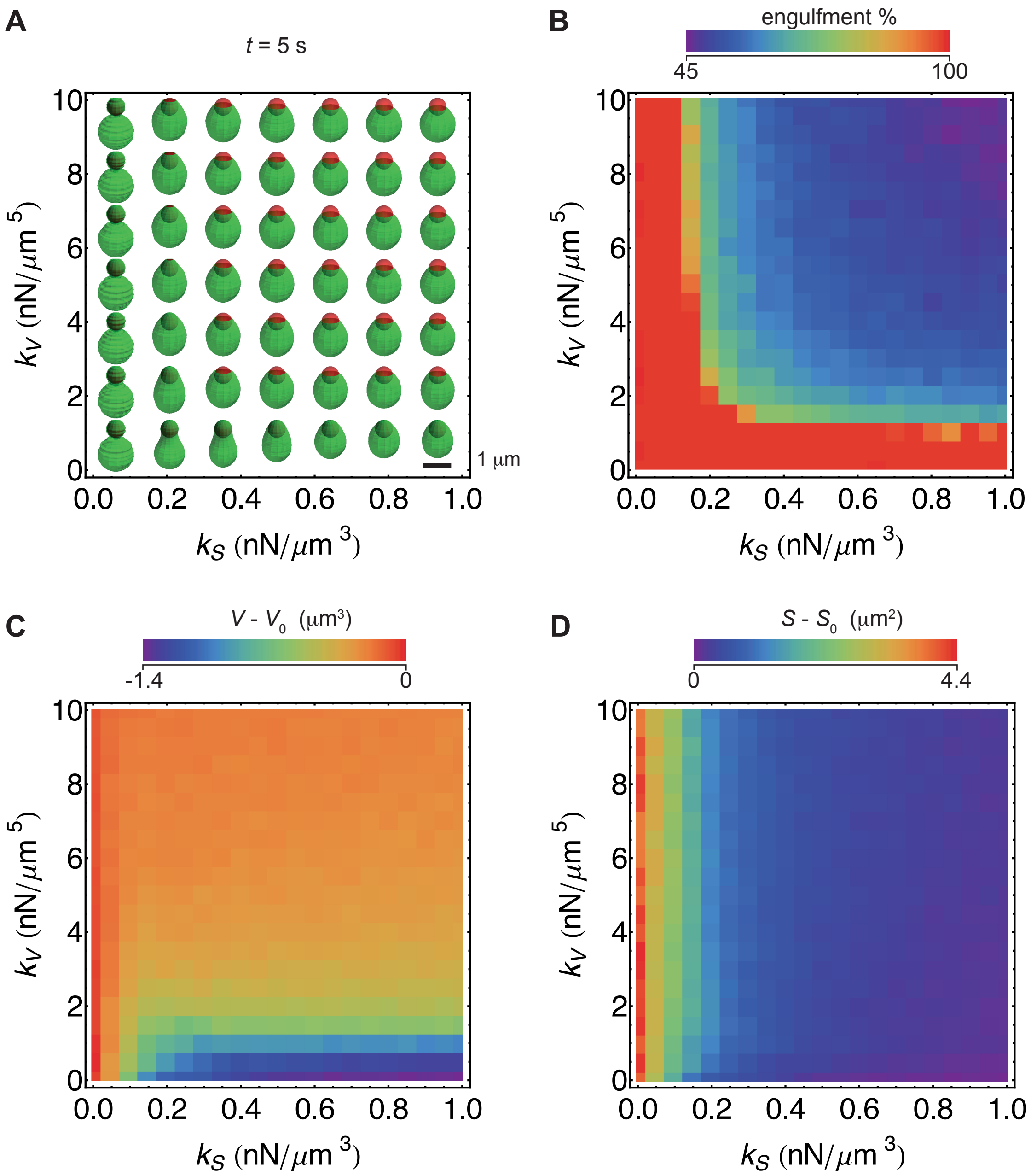

FIG. 8: **Simulation results of engulfment, volume, and surface area changes for a model with effective constraints.** (A) Simulation snapshots at 5 s for fixed SpoIIQ-SpoIIIAH surface density $\rho = 10^4 \mu\text{m}^{-2}$ as in Fig. 4 of the main text for different combinations of surface constraint parameter (k_S) and volume constraint parameter (k_V). Simulations that reached full engulfment earlier than 5 s were terminated and last snapshots are displayed only. (B) Percentage of forespore-surface area enclosed by mother membrane. In dark red region full engulfment occurs when either volume or surface area is not conserved. (C and D) Volume and surface area of mother cell at late stage of engulfment. The region of parameter space with experimentally observed volume and surface area changes occur is around: $k_S = (0.10 \pm 0.05)$ nN/μm^3 and $k_V = (1.0 \pm 0.5)$ nN/μm^5.

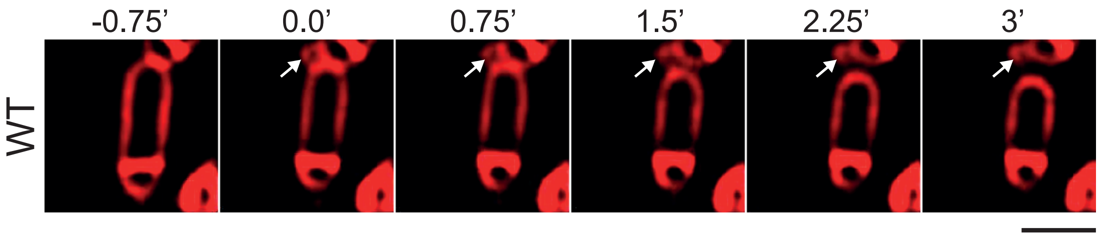

FIG. 9: **Cell loosing cytosol at onset of volume loss.** A montage of single WT cell treated with cell-wall removal enzyme, bursts (arrow) and cytosolic leakage coincide with the onset of volume loss (set to 0 min). Scale bar: 2 μm. Adopted from [2].

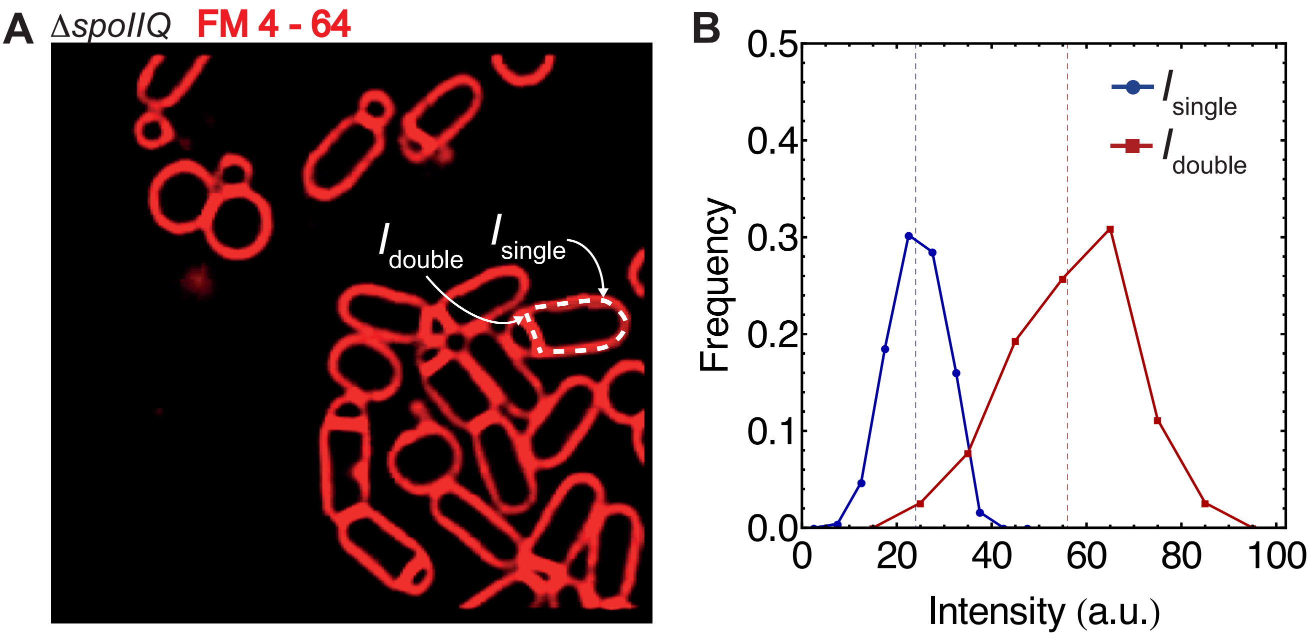

FIG. 10: **Test of fluorescence ratio with single and double membranes.** (A) FM 4-64 fluorescence intensities of membrane is measured along single ($I_{\rm single}$) and double $I_{\rm double}$ membranes. For this purpose we used Δ*spoIIQ* cells at late times after lysozyme addition when engulfing cups are flattened. (B) Histogram of intensities of all single and double membranes from panel (A). $I_{\rm double}/I_{\rm single} = 2.3 \pm 0.6$. Dashed vertical lines represent corresponding averages.

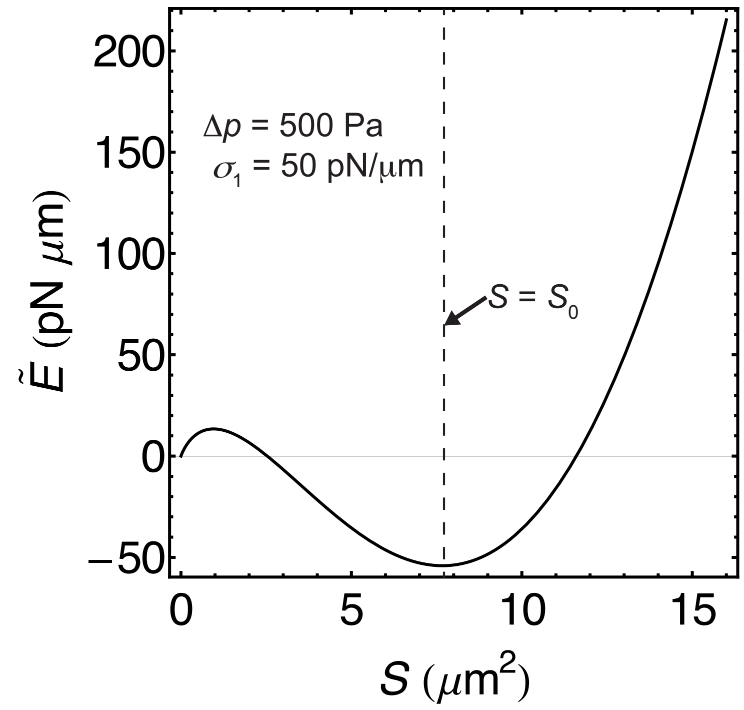

FIG. 11: **Energy vs surface area in model with energy balancing.** Energy $\tilde{E}$ is plotted as a function of surface area (see Eq. 15 with $V_0 = S_0^{\frac{3}{2}}/(6\sqrt{\pi})$). Parameters used: Δp= 500 Pa, σ_1 = 50 pN/μm, and σ_2 is calculated using Eq. 17.

[1] Smith MB, Li H, Shen T, Huang X, Yusuf E, et al. (2010) Segmentation and tracking of cytoskeletal filaments using open active contours. Cytoskeleton 67: 693–705.
[2] Broder DH, Pogliano K (2006) Forespore engulfment mediated by a ratchet-like mechanism. Cell 126: 917-928.
[3] Derényi I, Jülicher F, Prost J (2002) Formation and interaction of membrane tubes. Phys Rev Lett 88: 238101-238101.
[4] Deuling HJ, Helfrich W (1976) The curvature elasticity of fluid membranes: A catalogue of vesicle shapes. J Phys France 37: 1335.
[5] Howard J (2001) Mechanics of motor proteins and the cytoskeleton. Sinauer Associates.
[6] Boal D (2002) Mechanics of the Cell. Camb Univ Press.
[7] Pasquali M, Morse DC (2002) An efficient algorithm for metric correction forces in simulations of linear polymers with constrained bond lengths. J Chem Phys 116: 1834-1838.
[8] Meisner J, Moran CP (2011) A LytM domain dictates the localization of proteins to the mother cell-forespore interface during bacterial endospore formation. J Bacteriol 193: 591-598.
[9] Herant M, Heinrich V, Dembo M (2006) Mechanics of neutrophil phagocytosis: experiments and quantitative models. J Cell Sci 119: 1903–1913.
[10] Discher DE, Boal DH, Boey SK (1998) Simulations of the erythrocyte cytoskeleton at large deformation. ii. micropipette aspiration. Biophys J 75.
[11] Peng Z, Li X, Pivkin IV, Dao M, Karniadakis GE, et al. (2013) Lipid bilayer and cytoskeletal interactions in a red blood cell. Proc Natl Acad Sci USA 110: 13356–13361.
[12] Pécréaux J, Döbereiner HG, Prost J, Joanny JF, Bassereau P (2004) Refined contour analysis of giant unilamellar vesicles. Eur Phys J E Soft Matter 13: 277-290.
[13] Lieber AD, Yehudai-Resheff S, Barnhart EL, Theriot JA, Keren K (2013) Membrane tension in rapidly moving cells is determined by cytoskeletal forces. Curr Biol 23: 1409–1417.
[14] Wirtz D (2009) Particle-tracking microrheology of living cells: principles and applications. Annu Rev Biophys 38: 301-326.
[15] Levdikov VM, Blagova EV, McFeat A, Fogg MJ, Wilson KS, et al. (2012) Structure of components of an intercellular channel complex in sporulating *Bacillus subtilis*. Proc Natl Acad Sci USA 109: 5441-5445.
[16] Meisner J, Maehigashi T, André I, Dunham CM, Moran CP (2012) Structure of the basal components of a bacterial transporter. Proc Natl Acad Sci USA 109: 5446-5451.
[17] Sackmann E (1996) Supported membranes: scientific and practical applications. Science 271: 43–48.
[18] Betz T, Lenz M, Joanny JF, Sykes C (2009) ATP-dependent mechanics of red blood cells. Proc Natl Acad Sci USA 106: 15320–15325.
[19] Duwe H, Kaes J, Sackmann E (1990) Bending elastic moduli of lipid bilayers: modulation by solutes. J Phys France 51: 945–961.
[20] Milner ST, Safran S (1987) Dynamical fluctuations of droplet microemulsions and vesicles. Phys Rev A 36: 4371.
[21] Méléard P, Faucon J, Mitov M, Bothorel P (1992) Pulsed-light microscopy applied to the measurement of the bending elasticity of giant liposomes. Europhys Lett 19: 267.
[22] Kalwarczyk T, Tabaka M, Holyst R (2012) Biologistics – diffusion coefficients for complete proteome of *Escherichia coli*. Bioinformatics 28: 2971–2978.
